# Subvastus Arthrotomy and Multifaceted Fixation in Medial Femoral Comminuted Hoffa's Fracture: A Case Report

**DOI:** 10.7759/cureus.59121

**Published:** 2024-04-27

**Authors:** Siddharth K Patel, Sohael Khan, Ashutosh Lohiya, Ajay Koushik, Hardik Patel

**Affiliations:** 1 Department of Orthopaedics, Jawaharlal Nehru Medical College, Datta Meghe Institute of Higher Education and Research, Wardha, IND

**Keywords:** locking reconstruction plate, herbert screws, cannulated screws, subvastus arthrotomy, femoral condyle, hoffa's fracture

## Abstract

In managing orthopedic trauma, Hoffa's fracture, a rare intra-articular fracture affecting the femoral condyle, presents a unique challenge. We report a case of a 45-year-old male patient who had a traumatic injury and complained of substantial knee discomfort and limited range of motion. The patient had a medial femoral comminuted Hoffa's fracture. Subvastus arthrotomy was employed to handle the fracture successfully, and then a locking reconstruction plate, Herbert screws, and 4 mm cannulated screws were used for precise reduction and fixation. At the one-year follow-up, the patient showed acceptable postoperative results, including recovered knee function and radiographic indications of fracture repair.

## Introduction

The Hoffa fracture is named after Hoffa, who first reported it in 1904; it is defined as a femoral condyle fracture in the coronal plane affecting one or both condyles [1]. As the fracture is intra-articular and given the chance of potential articular cartilage damage, Hoffa's fractures affecting the medial femoral condyle present unique difficulties. These fractures are challenging to diagnose and sometimes go undetected on anteroposterior X-rays because the unfractured condylar section of the femur may obscure the broken condyle [2]. Such injuries are uncommon in adults and even rarer in teenagers; 8.7-13% of distal femur fractures are caused by them [3]. Owing to the knee joint's physiological genu valgus, injuries to the lateral condyle occur more frequently [4]. A Hoffa fracture is mostly caused by a high-energy trauma. It is advised to use surgical intervention, anatomical reduction, and solid fixation to lower the risk of issues such as osteonecrosis, nonunion, and arthritis [5].

Studies have documented the use of a range of surgical techniques, including open reduction and internal fixation (ORIF) with different implant configurations, to treat Hoffa cracks. Patients with comminuted Hoffa fractures, metaphyseal fracture extension, or low bone quality might be managed with hybrid fixation methods, which incorporate the use of a plate and screws [6,7]. For Hoffa fractures, ORIF is recommended regardless of the degree of fracture displacement, to anatomically restore the articular surface [8]. We report a case of comminuted Hoffa's fracture in the medial femur, which was fixed with 4 mm cannulated screws, a locking reconstruction plate, and Herbert screws by using a subvastus arthrotomy approach.

## Case presentation

**Figure 1:**
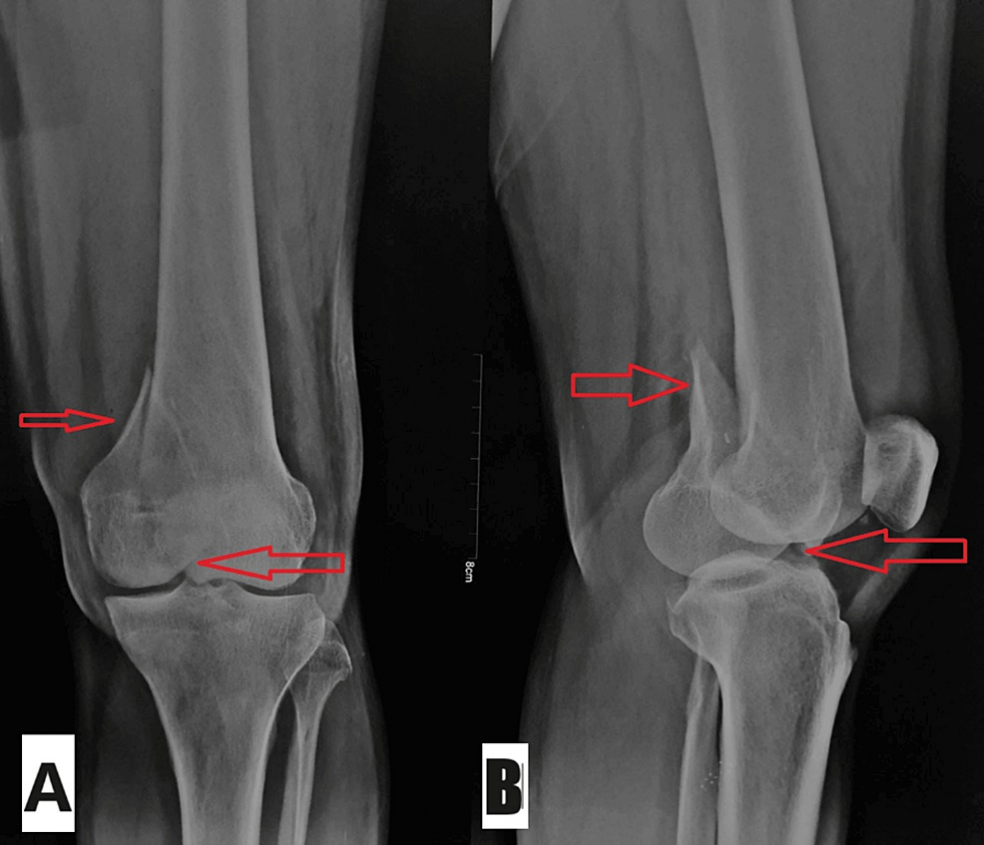
Preoperative X-ray of the left knee The anteroposterior (A) and lateral (B) views. The red arrows show medial femoral comminuted Hoffa's fracture with substantial displacement and joint involvement

The patient was a 45-year-old male who presented at our institution's emergency room with acute left knee pain and edema following a fall from a height. The initial examination revealed a limited range of motion, edema over the knee, discomfort over the medial femoral condyle, and evidence of an intra-articular fracture. The patient had no prior history of hospitalization and no known comorbidities. An X-ray of the left knee taken from the lateral and anteroposterior views validated the radiographic assessment of diagnosing a medial femoral comminuted Hoffa's fracture with significant displacement and joint involvement, as shown in Figure 1.

**Figure 2:**
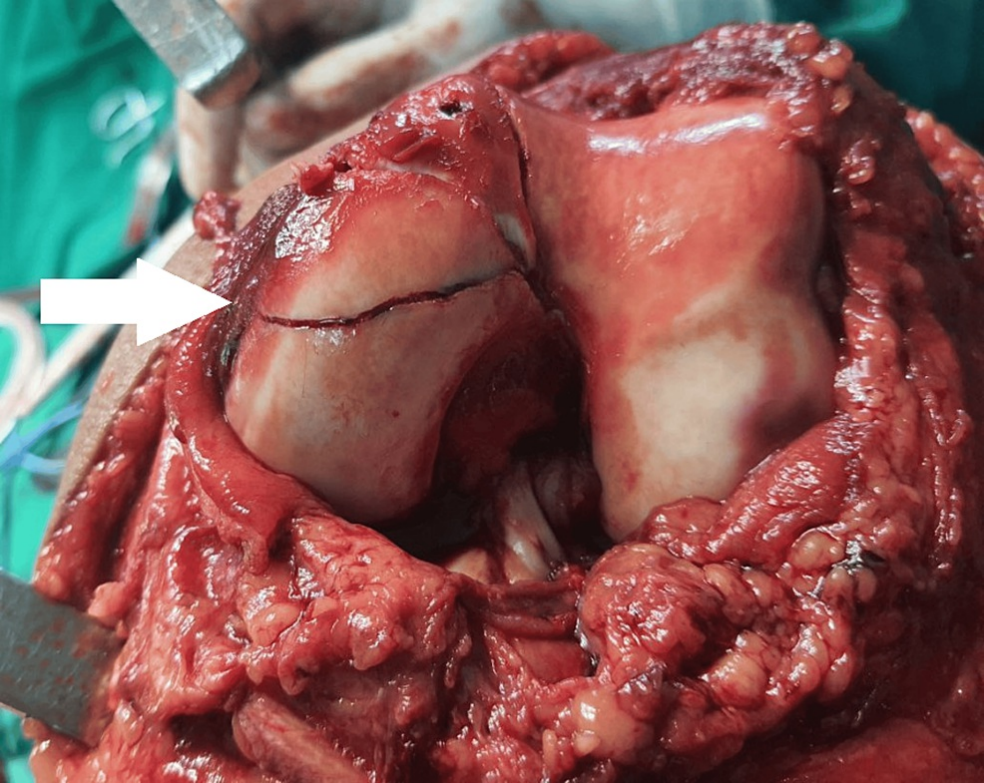
Intraoperative image - 1 The white arrow shows the fracture in the intra-articular surface of the medial femoral condyle

To stabilize the fracture, the patient was first treated with an above-knee slab, following which he was admitted. The patient was managed with a subvastus arthrotomy approach combined with fixation using 4 mm cannulated screws, Herbert screws, and a locking reconstruction plate. He was put under spinal anesthesia, put in a supine position on an operative theatre (OT) table, and had a tourniquet applied to the left thigh. The knee joint was accessed via a subvastus arthrotomy technique, which preserved the extensor mechanism and reduced soft tissue damage and fracture, as shown in Figure 2.

**Figure 3:**
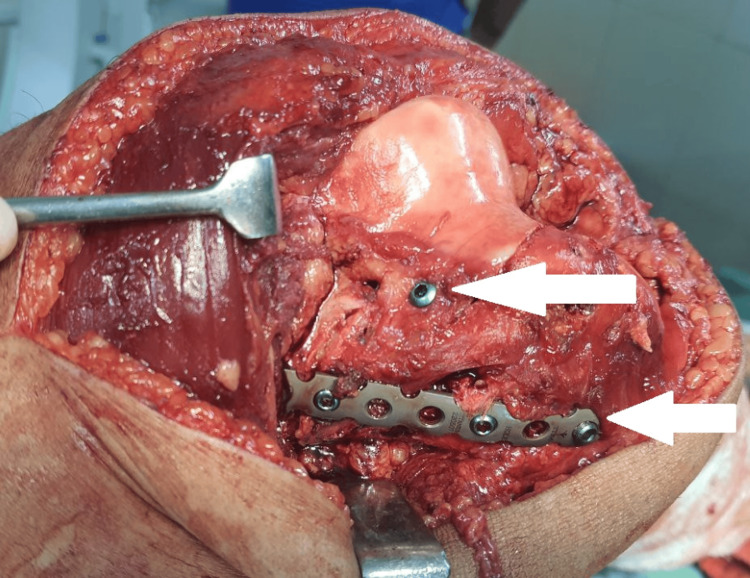
Intraoperative image - 2 The white arrow shows the fracture stabilized by a reconstruction plate and Herbert screws

**Figure 4:**
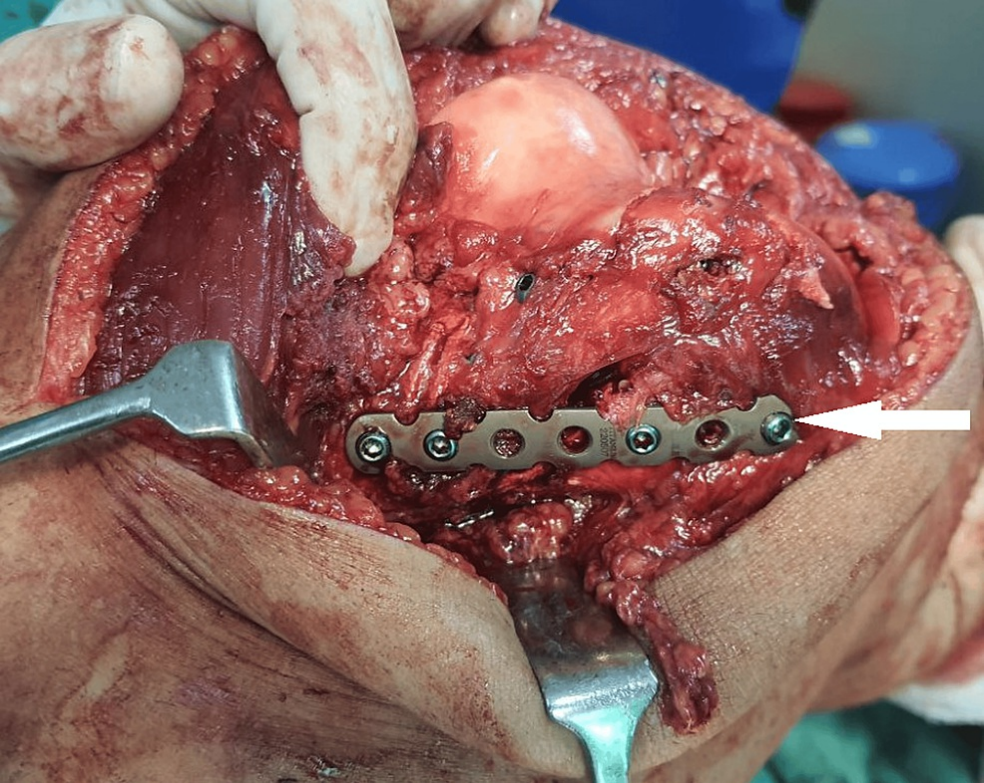
Intraoperative image - 3 The white arrow shows the fracture stabilized by a locking reconstruction plate and Herbert screws

The extent of the fracture was established by intraoperative fluoroscopy, and then anatomical reduction forceps and manual manipulation were used to reduce the fracture carefully. The fracture was then stabilized by applying a locking reconstruction plate in a buttress shape, 4 mm cannulated screws, and Herbert screws to support the comminuted pieces and restore joint congruity, as shown in Figure 3 and Figure 4.

**Figure 5:**
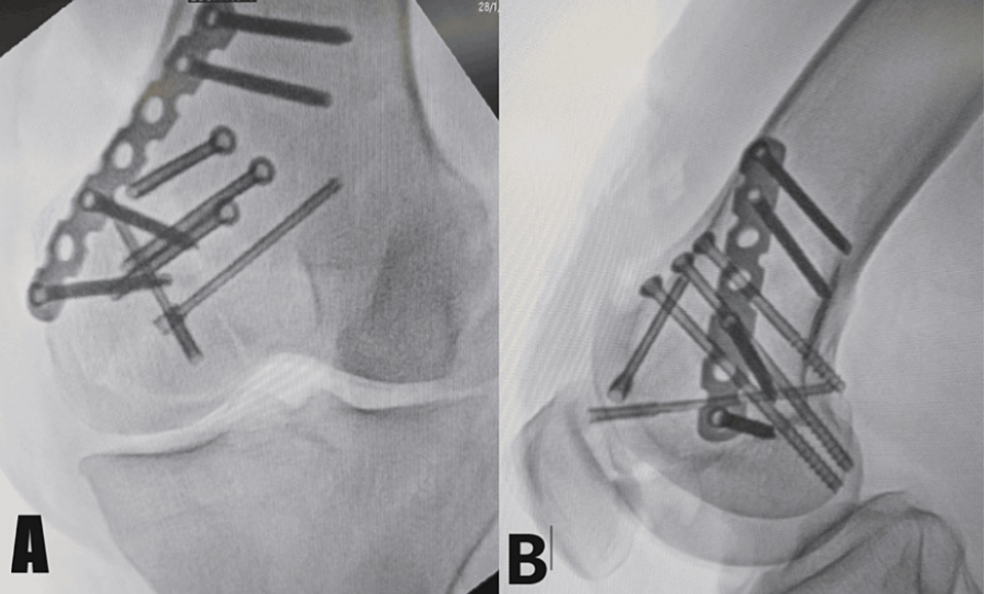
Postoperative X-rays Anteroposterior (A) and lateral (B) views show fracture reduction done by the locking reconstruction plate in a buttress shape, 4 mm cannulated screws, and Herbert screws

**Figure 6:**
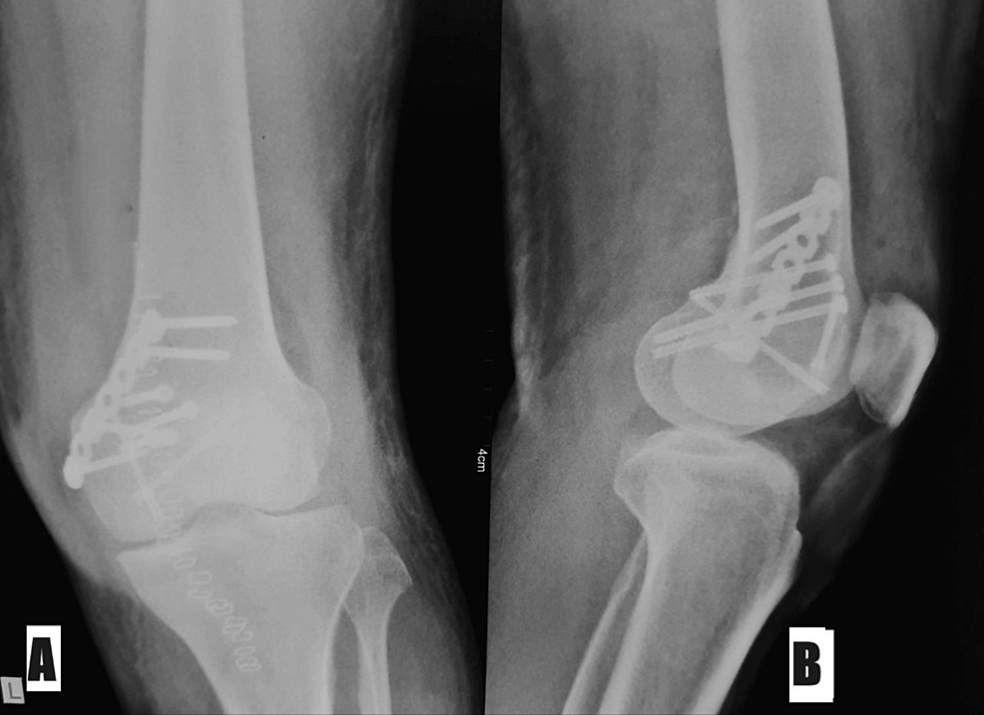
Follow-up postoperative X-ray at one year Anteroposterior (A) and lateral (B) views show the united fracture with a locking reconstruction plate in a buttress shape, 4 mm cannulated screws, and Herbert screws in situ

Following surgery, the patient followed a planned rehabilitation program that included crutch-assisted partial weight-bearing, range-of-motion exercises, and early mobilization. Regular radiographic evaluation showed increasing fracture healing and joint alignment restoration. At the one-year follow-up, the patient reported a significant improvement in knee function, minimal pain persistence, and a manageable range of motion. Signs of remodeling and fracture union were seen on radiographs, indicating a successful surgical intervention, as shown in Figure 5 and Figure 6.

## Discussion

A Hoffa's fracture is a complex intra-articular fracture involving the weight-bearing surface of the femoral condyle, also known as a coronal fracture of the femoral condyle. These fractures usually result from high-energy trauma from car crashes or falls from considerable heights. These fractures are complicated due to their locations and how the injury was sustained, potentially making treatment difficult and raising the possibility of adverse effects. A major problem with Hoffa's fractures is the increased risk of complications like nonunion, malunion, and avascular necrosis. It is rare to have a medial femoral condyle coronal fracture in isolation, leaving the lateral condyle intact [9]. Given the possibility of articular incongruity, posttraumatic arthritis, and functional impairment, Hoffa's fractures remain a challenge to treat.

No surgical approach or fixing technique has been generally acknowledged to provide the best results in these patients [10]. Nonetheless, several strategies have been used to treat these intricate fractures. Although there is a chance of neurovascular damage, direct lateral/medial and posterolateral methods have the benefit of improved visualization and access to smaller Hoffa fragments. To offer even more stability, a plate may occasionally be utilized in conjunction with posterior-anterior direction screws [11].

​​​The subvastus arthrotomy approach was used in our case to minimize soft tissue disturbance and provide sufficient exposure to the fracture site. This method, along with the use of Herbert screws, 4 mm cannulated screws, and a locking reconstruction plate, allowed for early mobilization and stable fixation, leading to favorable clinical outcomes. The subvastus arthrotomy and combination of screws and locking reconstruction plate utilized in this case's surgical technique allowed for sufficient exposure and solid fixation, eventually resulting in positive results.

## Conclusions

Medial femoral comminuted Hoffa's fractures are uncommon but challenging orthopedic injuries that need to be recognized promptly and treated surgically if required. Subvastus arthrotomy offers a viable therapeutic option with the potential for good clinical results and fracture union when paired with fixation utilizing 4 mm cannulated screws, Herbert screws, and a locking reconstruction plate. More research is necessary to assess this method's long-term effects in treating Hoffa's fractures.
